# The efficacy and safety of adjuvant immunotherapy after neoadjuvant immunotherapy combined with chemotherapy in locally advanced resectable esophageal squamous cell carcinoma: a real−world study

**DOI:** 10.3389/fimmu.2025.1555756

**Published:** 2025-05-21

**Authors:** Xue Wu, Fangjie Ding, Shunshun Bao, Yiming Mu, Jiandong Zhang, Shuming Zhang, Pingping Hu, Yan Zhang, Ning Liang, Guodong Deng, Yuying Hao, Xinquan Liang, Fengxue Li, Yang Shu, Jingxin Zhang, Lili Qiao, Yingying Zhang

**Affiliations:** ^1^ Department of Oncology, The First Affiliated Hospital of Shandong First Medical University & Shandong Provincial Qianfoshan Hospital, Jinan, Shandong, China; ^2^ Clinical Medical College of Jining Medical University, Jining, Shandong, China; ^3^ Department of Radiation Oncology, Shandong Cancer Hospital and Institute, Shandong First Medical University and Shandong Academy of Medical Sciences, Jinan, Shandong, China; ^4^ Shandong University Cancer Center, Shandong University, Jinan, Shandong, China; ^5^ The Second Clinical Medical College, Shandong University of Traditional Chinese Medicine, Jinan, Shandong, China; ^6^ Cheeloo College of Medicine, Shandong University, Jinan, China

**Keywords:** adjuvant therapy, neoadjuvant therapy, immunotherapy, chemotherapy, esophageal squamous cell carcinoma (ESCC)

## Abstract

**Background:**

The promising therapeutic outcomes of neoadjuvant immunotherapy with chemotherapy (NAIC) in the treatment of resectable locally advanced esophageal squamous cell carcinoma (LA-ESCC) have been confirmed by several clinical trials. However, the potential benefits of adjuvant therapy for LA-ESCC patients remain unclear.

**Materials and methods:**

We analyzed the LA-ESCC patients underwent NAIC and adjuvant immunotherapy between January 2020 and September 2023. The effectiveness and feasibility of adjuvant immunotherapy were evaluated.

**Results:**

A total of 112 LA-ESCC patients were included. With a median follow-up of 24.0 months, all 112 patients had an R0 resection, and 23 patients (20.5%) achieved pathological complete response (pCR). The median disease-free survival (DFS) and overall survival (OS) were 18.5 and 24.0 months. The 12- and 24-month DFS rates were 91.0% and 81.7%, and the 12- and 24-month OS rates were 99.1% and 96.8%, respectively. Patients with BMI ≥20 kg/m^2^ had a longer 24-month DFS rate compared with those with BMI <20 kg/m^2^ (87.1% vs 62.0%, P=0.034). Additionally, patients with postoperative pCR than those with non-pCR achieved better 12-month (100% vs 88.6%) and 24-month (100% vs 77.3%, P<0.001) DFS. Superior DFS rates were acquired in patients with ypT0-1 (12-month: 98.1% vs 84.6%, P=0.008, 24-month: 95.4% vs 70.7%, P<0.001), ypN0 (12-month: 96.9% vs 83.1%, P=0.019, 24-month: 88.9% vs 72.2%, P=0.042), obtained T (12-month: 96.2% vs 78.3%, P=0.018, 24-month: 92.8% vs 56.0%, P<0.001) or TNM (12-month: 96.5% vs 84.8%, P=0.033, 24-month: 90.5% vs 72.5%, P=0.02) downstaging. A total of 85 (78.0%) patients experienced treatment-related adverse events (TRAEs), with the most common TRAEs were digestive reactions (52.3%) and neutropenia (50.4%). The majority of these events were classified as grade 1-2.

**Conclusion:**

The combination of NAIC and adjuvant immunotherapy displays short survival benefits and has an acceptable safety profile, which may be an effective treatment strategy for LA-ESCC patients.

## Introduction

Esophageal cancer (EC) ranked as the seventh most common cancer and the sixth leading cause of cancer-related death in 2020 (1). The predominant histopathological type is squamous cell carcinoma, particularly in China (2, 3). Over two-thirds of patients with esophageal squamous cell carcinoma (ESCC) are diagnosed at a locally advanced stage (2). Surgical resection with curative intent remains the main treatment for locally advanced ESCC (LA-ESCC). Based on the CROSS and NEOCRTEC5010 trials, neoadjuvant chemoradiotherapy (NCRT) has become the standard treatment for resectable LA-ESCC (4, 5). However, approximately 35% of patients still experienced tumor recurrence, and faced a high risk for death (6, 7). Consequently, it is essential to explore novel treatment strategies for resectable LA-ESCC to reduce the risk of recurrence and improve prognosis.

Immune checkpoint inhibitors (ICIs) have revolutionized the treatment profile of ESCC. The combination of ICIs and chemotherapy has shown promising efficacy and safety in advanced ESCC and is now recommended as a first-line treatment, as demonstrated by the KEYNOTE-590, RATIONALE-306, and CheckMate 648 trials (8–12). However, the application of ICIs in LA-ESCC is still in the exploratory stage, particularly in perioperative treatment. Recently, the ESCORT-NEO/NCCES01 trial revealed that neoadjuvant ICIs combined with chemotherapy compared with chemoradiotherapy significantly increased the pathological complete response (pCR) rate in the LA-ESCC, without a significant increase in toxicity (13). Similarly, the REVO study confirmed that the pCR rate of neoadjuvant immunotherapy combined with chemotherapy (NAIC) was higher than that with concurrent chemoradiotherapy (14). These studies provide evidence for NAIC as an effective perioperative therapeutic strategy for resectable LA-ESCC, but whether patients can further benefit from adjuvant immunotherapy has not been clarified.

In this study, we evaluated the efficacy and safety of NAIC followed by surgical resection and adjuvant immunotherapy in patients with LA-ESCC, aiming to provide support for the clinical application of this new strategy.

## Methods

### Participants

**Figure 1:**
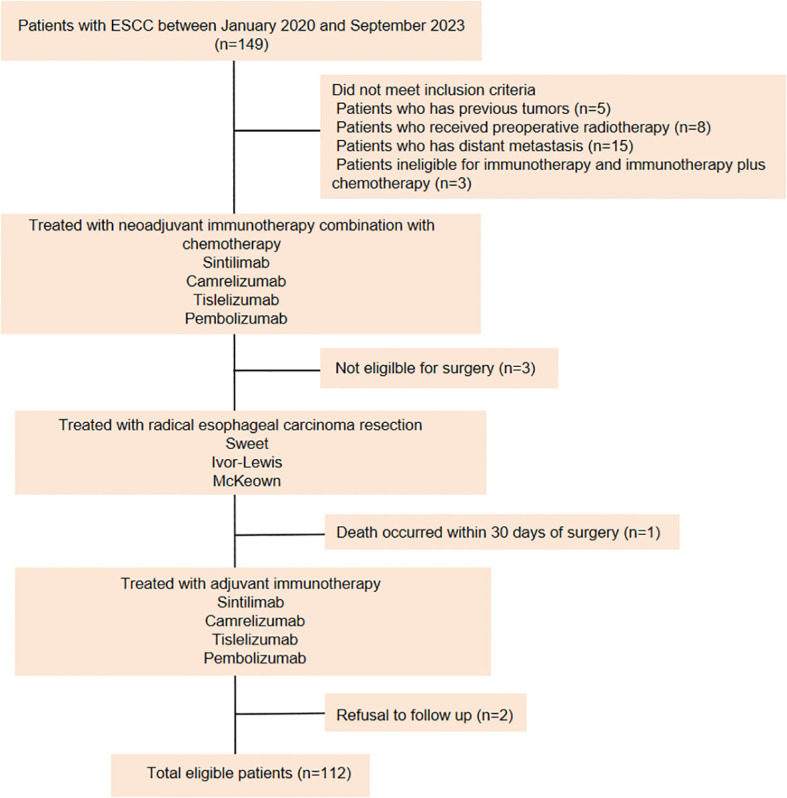
Flow chart. ESCC, esophageal squamous cell carcinoma.

Data from 112 LA-ESCC patients who underwent NAIC followed by adjuvant immunotherapy between January 2020 and September 2023 at the First Affiliated Hospital of Shandong First Medical University (Shandong Provincial Qianfoshan Hospital) and the Affiliated Cancer Hospital of Shandong First Medical University (Shandong Cancer Hospital) were retrospectively analyzed (**Figure 1**). Patients were eligible for enrollment if they met the following criteria: (a) histopathologically confirmed ESCC diagnosis, (b) completion of NAIC followed by surgery, (c) receipt of adjuvant immunotherapy or a combination of immunotherapy with radiotherapy or chemotherapy after surgery, and (d) Karnofsky Performance Status (KPS) score between 70 and 100, with no severe dysfunction in major organs (cardiac, pulmonary, hepatic, renal, or hematologic). The exclusion criteria were as follows: (a) patients with previous tumors; (b) patients underwent preoperative radiotherapy; (c) patients who have distant metastasis; (d) patients ineligible for immunotherapy or immunotherapy plus chemotherapy, and (e) patients who refused follow-up. Data were collected through retrospective chart review. The variables included age, sex, BMI, KPS score, smoking history, drinking history, concomitant diseases, family history, neoadjuvant and adjuvant treatment regimens, surgical methods, tumor location, histologic subtype, clinical and pathologic diagnosis of TNM stage according to American Joint Committee on Cancer (AJCC) stage (8th edition staging manual). This study was approved by the institutional ethical review board (study numbers 2022 S398 and SDTHEC202409044). Due to the retrospective design, the Committee agreed to give up the written informed consent.

### Procedures

Patients were staged using endoscopic ultrasound (EUS), computerized tomography (CT) or positron emission tomography-CT (PET-CT), or magnetic resonance imaging (MRI) before neoadjuvant therapy. Adjuvant therapy includes combinations of immunotherapy with chemotherapy and/or radiotherapy. NAIC consisted of 2 to 4 cycles of ICIs, primarily camrelizumab, sintilimab, pembrolizumab, or tislelizumab (200 mg every 3 weeks), combined with chemotherapy. The chemotherapy regimens included paclitaxel (albumin-bound) with a platinum-based drug (e.g., nedaplatin, cisplatin, or carboplatin), paclitaxel (albumin-bound) with fluorouracil (tegafur or 5-fluorouracil), or paclitaxel (albumin-bound) with both a platinum-based drug and tegafur. Multidisciplinary consultation was conducted to determine whether patients should proceed with surgery or continue treatment. Esophagectomy was generally performed 4 to 6 weeks after the completion of neoadjuvant therapy. All patients underwent standard radical surgery for LA-ESCC. Most patients began adjuvant immunotherapy within 4-8 weeks post-operation, with adjuvant immunotherapy and chemotherapy regimens corresponding to the neoadjuvant treatment. Some patients received concurrent or sequential radiotherapy with doses of 45 to 50 Gy.

### Outcomes

The primary endpoints were disease-free survival (DFS) and overall survival (OS). Secondary endpoints included the pCR rate, surgical complications and adverse events. Adverse events during adjuvant therapy were assessed according to the National Cancer Institute’s Common Terminology Criteria for Adverse Events (version 5.0). DFS was interval between esophagectomy and the last follow-up visit, recurrence, distant metastasis, or death from any cause. OS was the time from initial diagnosis to final follow-up or death from any cause during follow-up. R0 resection was defined as the complete removal of tumors with negative microscopic margins. The pCR refers to the absence of residual tumor cells at the primary site in the surgical sample and in all lymph nodes removed from the surgical specimen (ypT0N0). The pCR rate was calculated as the number of patients with pCR divided by the total number of evaluable patients. Downstaging was defined as a reduction in pathologic staging after NAIC compared with baseline. Follow-up data were collected using electronic medical records and telephone interviews. All patients were regularly followed up until mortality or the last visit on September 30, 2024. Subgroup analysis was conducted to evaluate factors affecting DFS and OS. The Combined Positive Score (CPS) was defined as the percentage of PD-L1-positive tumor cells and immune cells (lymphocytes and macrophages) divided by the number of viable tumor cells multiplied by 100.

### Statistical analysis

All statistical analyses were performed using R software (version 4.2.1) and SPSS 26.0. Categorical variables were presented as frequencies and percentages. OS and DFS were analyzed using the Kaplan-Meier method, and hazard ratios (HR) with 95% confidence intervals (CI) calculated. The Kaplan-Meier estimator was used to plot survival functions over time, with the Greenwood formula applied to estimate standard errors at specific time points, namely at 12 and 24 months, to assess the variability in survival rate estimates. The statistical significance of differences in survival between groups was evaluated by the log-rank test. Bilateral P-values were considered statistically significant when they were less than 0.05. Survival plots were generated using the ‘ggsurvplot’ function from the ‘survminer’ package in R, with confidence intervals included for each group. Annotations for survival rates, HR, and P-values at 12 and 24 months were added to the Kaplan-Meier plots for clarity.

## Results

### Patient baseline and characteristics

Between January 2020 and September 2023, a total of 112 patients with LA-ESCC who received NAIC and adjuvant immunotherapy were enrolled in the study. The clinical characteristics of these patients are summarized in **Table 1**. At data cutoff (September 30, 2024), the median duration of follow-up (time from initial diagnosis to the cutoff date) was 24.0 months. Of the 112 patients, 99 (88.4%) were male and 13 (11.6%) were female, 70 (62.5%) were <65 years old, and 42 (37.5%) were ≥65 years old. Among these patients, 27 (24.1%) patients had a BMI <20 kg/m^2^, and the rest (75.9%) patients exhibited a BMI ≥20 kg/m^2^. The majority of patients had a history of smoking and drinking. Before being diagnosed with LA-ESCC, 61 (54.5%) patients were otherwise healthy, while the remaining patients had underlying conditions, with hypertension being the most common. At diagnosis, 99 (88.4%) patients had clinical T3 disease, 78 (69.6%) had lymph node involvement (N+), and 77 (68.8%) were classified as stage III. Tumors were located at the upper (2.7%), middle (38.4%), and lower (58.9%) thoracic esophagus. Furthermore, baseline expression of PD-L1 in tumor microenvironment (TME) was categorized into two groups according to CPS. The results showed that 18 (16.1%) of patients had PD-L1 CPS<10, while 12 (10.7%) of patients displayed PD-L1 CPS≥10.

**Table 1:** Clinical characteristics of patients.

Variables	N =112
Age (%)	
<65	70 (62.5)
≥65	42 (37.5)
Gender (%)	
Male	99 (88.4)
Female	13 (11.6)
BMI kg/m2 (%)	
<20	27 (24.1)
≥20	85 (75.9)
KPS score (%)	
70	1 (0.9)
80	67 (59.8)
90	44 (39.3)
Smoking history (%)	
No	34 (30.4)
Yes	78 (69.6)
Drinking history (%)	
No	40 (35.7)
Yes	72 (64.3)
Comorbidity (%)	
No	61 (54.5)
Yes	51 (45.5)
Family history (%)	
No	99 (88.4)
Yes	13 (11.6)
Tumour location (%)	
Upper	3 (2.7)
Middle	43 (38.4)
Lower	66 (58.9)
Tumor grade (%)	
Well-differentiated	24 (21.4)
Moderately differentiated	62 (55.4)
Poorly differentiated	6 (5.4)
Unknown	20 (17.9)
Clinical T stage (%)	
T1	0 (0)
T2	8 (7.1)
T3	99 (88.4)
T4	5 (4.5)
Clinical N stage (%)	
N0	34 (30.4)
N+	78 (69.6)
Clinical TNM stage (%)	
II	31 (27.7)
III	77 (68.8)
IVa	4 (3.6)
PD-L1 CPS (%)	
<10	18 (16.1)
≥10	12 (10.7)
Not evaluable	82 (73.2)

BMI, body mass index; KPS, Karnofsky Performance status; TNM, tumour node metastasis; CPS, Combined Positive Score.

### Treatment outcomes

Of those patients, more than 50% were treated with camrelizumab combined with paclitaxel (albumin bound) and a platinum-based drug in neoadjuvant therapy and adjuvant therapy. The detailed information is listed in **Table 2**. In the neoadjuvant therapy phase, 83 (74.1%) patients chose camrelizumab as an immunotherapy agent, and 25 (22.3%) patients used sintilimab. 89 (79.5%) patients underwent surgery after 2 cycles of treatment. Radical esophageal carcinoma resection was performed in 83.7% of patients 4 to 6 weeks after neoadjuvant therapy. Surgical and postoperative pathological information of patients are summarized in **Table 3**. R0 resection was achieved in all the patients. McKewon (83.0%) and Ivor-Lewis (14.3%) surgeries were performed on the patients, and only three patients underwent Sweet operation. 23 (20.5%) patients acquired pCR. Pathologic T0-1 stage was achieved in 47.3% of patients, and pathologic N0 stage was gained in 64 patients (57.1%). 59 (52.7%) patients acquired clinical to pathological TNM downstaging. Furthermore, 79 (70.5%) patients had downstaging of T status, and 49 (43.8%) patients had the N downstaging. 100 (89.3%) patients underwent adjuvant immunotherapy over 4 weeks after surgical resection. No more than 2 cycles of ICIs were received by 62 (55.4%) patients. The immunotherapy regimens were applied in 82 (73.2%) patients with camrelizumab, 27 (24.1%) patients with sintilimab, 3 (2.7%) patients with tislelizumab, and 1 (0.9%) patient with pembolizumab. In this study, 104 (92.9%) patients were treated with chemotherapy combined with immunotherapy as adjuvant treatment, and 18 (16.1%) patients underwent concurrent or sequential radiotherapy with doses of 45 to 50 Gy.

**Table 2:** The information of neoadjuvant and adjuvant the therapeutic model.

Variables	Neadjuvant (N =112)	Adjuvant (N =112)
Neoadjuvant immunotherapeutic regimen (%)		
Sintilimab	25 (22.3)	27 (24.1)
Camrelizumab	83 (74.1)	82 (73.2)
Pembolizumab	1 (0.9)	1 (0.9)
Tislelizumab	3 (2.7)	2 (1.8)
Immunotherapeutic cycle (%)		
≤2	89 (79.5)	62 (55.4)
>2	16 (14.3)	50 (44.6)
Interval to surgery [weeks] (%)		
≤4	15 (13.4)	12(10.7)
>4	97 (86.6)	100 (89.3)
Combination treatment options (%)		
Chemotherapy	112 (100)	104 (92.9)
Radiotherapy	0 (0)	18 (16.1)
Chemoradiation	0 (0)	17 (15.2)
Immunotherapy only	0 (0)	7 (6.3)

**Table 3:** The operative information and postoperative pathological information of patients.

Parameters	N =112
Surgery excision method (%)	
Sweet	3 (2.7)
Ivor Lewis	16 (14.3)
McKewon	93 (83.0)
pCR (%)	
No	89 (79.5)
Yes	23 (20.5)
Pathological T stage (%)	
T0-1	53 (47.3)
T2-4	59 (52.7)
**Pathological N stage (%)**	
N0	64 (57.1)
N+	48 (42.9)
Pathological TNM stage (%)	
I-II	63 (56.3)
III-IV	49 (43.8)
Downstaging of T stage (%)	
No	33 (29.5)
Yes	79 (70.5)
Downstaging of N stage (%)	
No	63 (56.3)
Yes	49 (43.8)
Downstaging of TNM stage (%)	
No	53 (47.3)
Yes	59 (52.7)
Adverse events (%)	
Pneumonia	50 (45.9)
Pleural effusion	64 (58.7)
Pneumothorax	3 (2.8)
Recurrent laryngeal nerve injury	1 (0.9)
Anastomotic fistula	11 (10.2)
Anastomotic stricture	2 (1.8)

pCR, pathological complete response; TNM, tumour node metastasis.

### Survival

**Figure 2:**
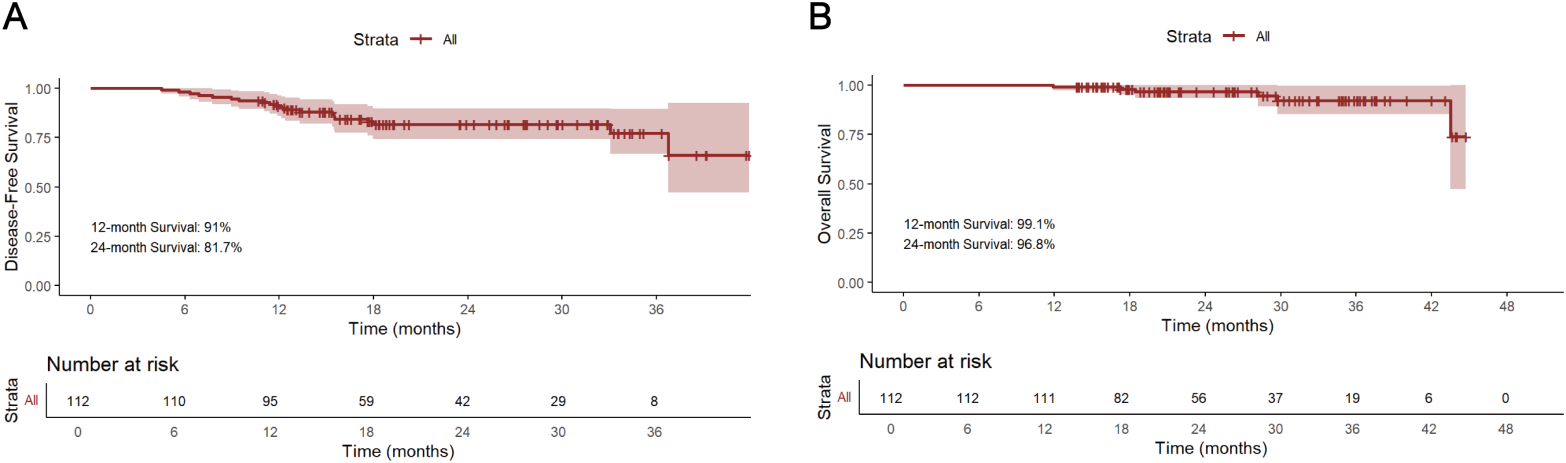
Kaplan-Meier survival analysis of DFS **(A)** and OS **(B)**. DFS, disease-free survival; OS overall survival.

**Figure 3:**
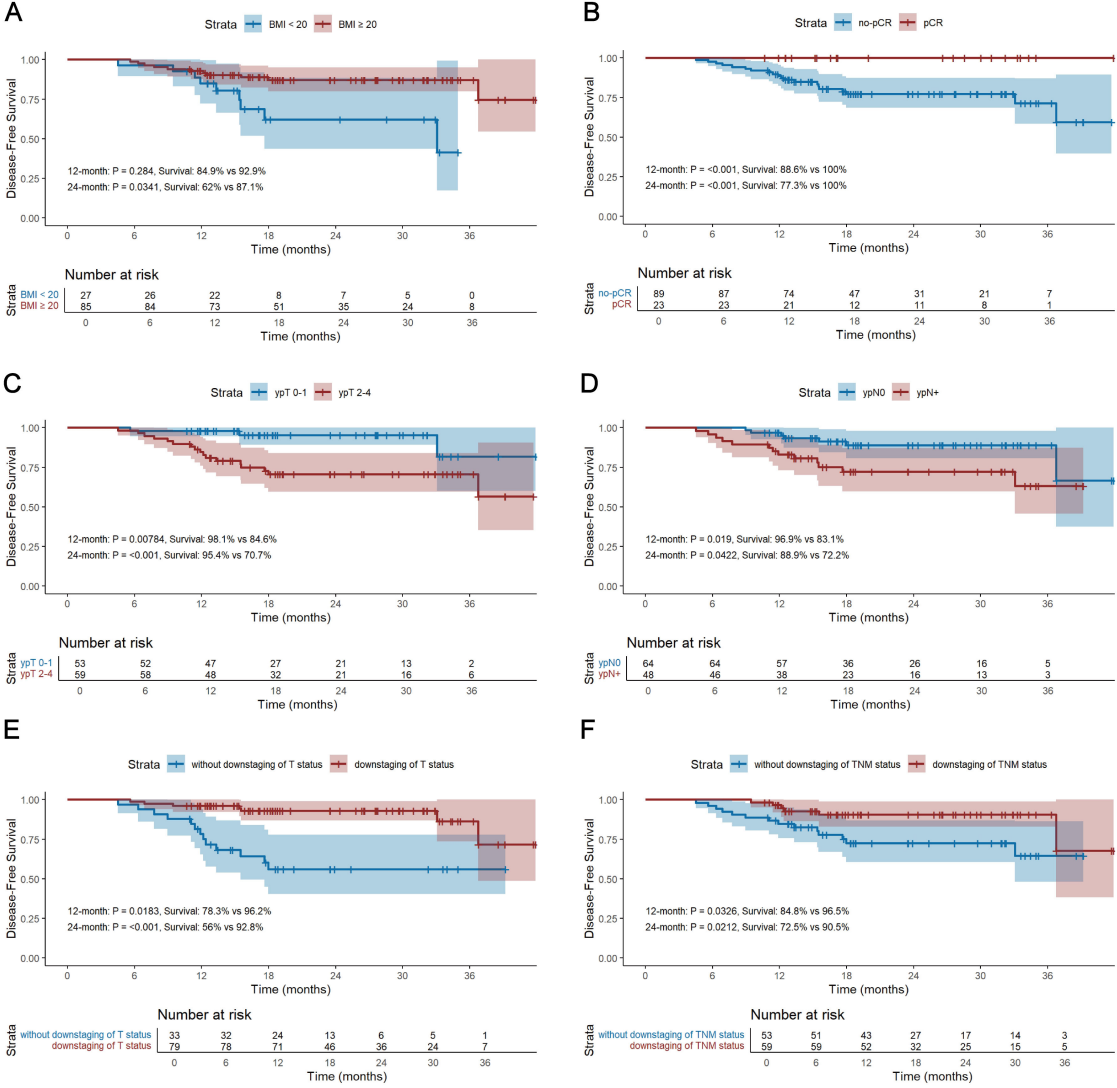
Kaplan-Meier survival analysis of DFS between BMI ≥20 kg/m^2^ and BMI <20 kg/m^2^ **(A)**, between pCR and non-pCR **(B)**, between ypT0-1 and ypT2-4 **(C)**, between ypN0 and ypN+ **(D)**, between T downstaging and without T downstaging **(E)**, and between TNM downstaging and without TNM downstaging **(F)**. DFS, disease-free survival; OS, overall survival; BMI, body mass index; pCR, pathological complete response.

**Figure 4:**
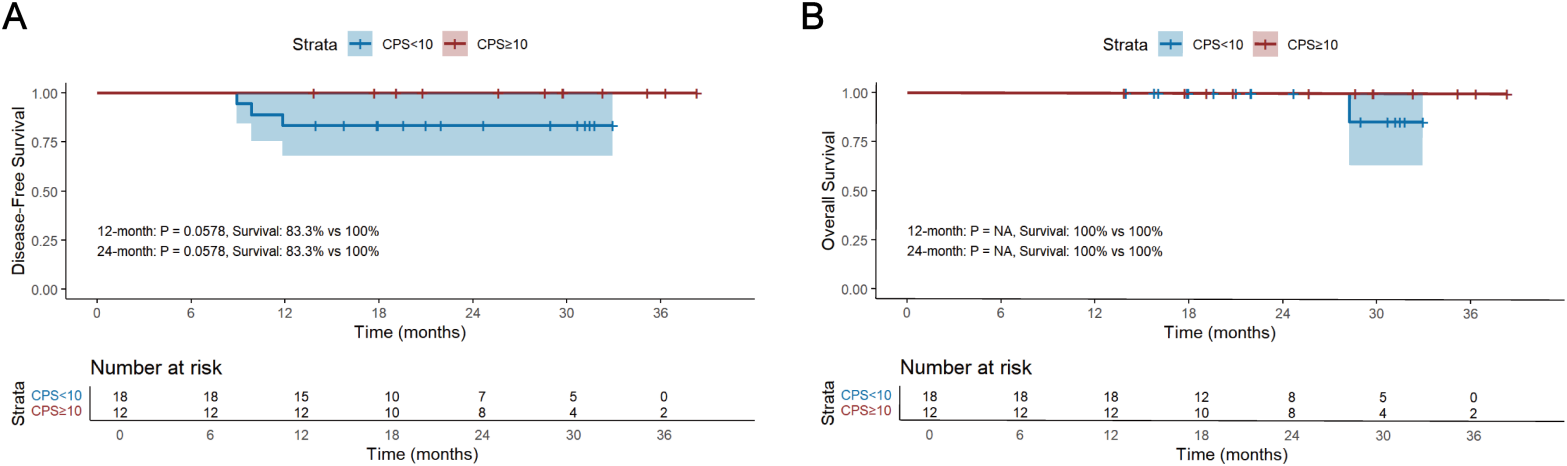
Kaplan-Meier survival analysis of DFS **(A)** and OS **(B)** and between PD-L1 CPS ≥10 and PD-L1 CPS <10. DFS, disease-free survival; OS, overall survival; CPS, the Combined Positive Score.

The median follow-up period for survivors was 24.0 months, ranging from 13.8 to 45.0 months, with a data cut-off date of September 2024. Median DFS and OS were 18.5 months and 24.0 months, respectively (**Figure 2**). The 12-month DFS and OS rates were 91.0% and 99.1%. While the 24-month DFS and OS rates were 81.7% and 96.8%. Further subgroup analysis found that patients with BMI ≥20 kg/m^2^ had a significantly better 24-month DFS rate compared with those BMI <20 kg/m^2^ (87.1% vs 62.0%, P=0.034, **Figure 3A**). Additionally, patients with postoperative pCR than those with non-pCR achieved better DFS rates both in 12-month (100% vs 88.6%, P<0.001) and 24-month (100% vs 77.3%, P<0.001, **Figure 3B**). Superior 12- and 24-month DFS rates were acquired in patients with ypT0-1 versus ypT2-4 (12-month: 98.1% vs 84.6%, P=0.008, 24-month: 95.4% vs 70.7%, P<0.001, **Figure 3C**), and ypN0 versus ypN+ (12-month: 96.9% vs 83.1%, P=0.019, 24-month: 88.9% vs 72.2%, P=0.042, **Figure 3D**). Furthermore, better 12- and 24-month DFS rates were shown in patients with T (12-month: 96.2% vs 78.3%, P=0.018, 24-month: 92.8% vs 56.0%, P<0.001, **Figure 3E**) and TNM (12-month: 96.5% vs 84.8%, P=0.033, 24-month: 90.5% vs 72.5%, P=0.021, **Figure 3F**) downstaging. However, patients with BMI ≥ 20 kg/m^2^, pCR, ypT0-1, ypN0, T and TNM downstaging did not show better OS benefits (**Supplementary Figure S1**). Additionally, Kaplan-Meier survival analyses of DFS and OS regarding clinical stage and clinical/pathologic nodal status are shown in **Supplementary Figure S2**. We found those were not associated with survival benefit. No difference in OS and DFS was observed between immunotherapy cycles (**Supplementary Figure S3**) and surgical intervals (**Supplementary Figure S4**) in either neoadjuvant or adjuvant phases. As shown in **Supplementary Figure S5**, no significant improvement in DFS or OS was observed with immunotherapy, either alone or in combination with radiotherapy or chemotherapy. Notably, patients with PD-L1 CPS ≥10 had a trend toward better 12-month and 24-month DFS rates than those with PD-L1 CPS <10 (100% vs 83.3%, P=0.058, **Figure 4**).

### Treatment-related adverse events

We evaluated detailed treatment information from 109 of the 112 patients. Three patients who received adjuvant immunotherapy at other hospitals were excluded, so the detailed information was not available. Treatment-related adverse events (TRAEs) were reported in 85 patients (78.0%), with the majority of events was classified as grade 1-2. including gastrointestinal reactions (52.3%) and neutropenia (50.4%). In total, 66 patients (60.6%) experienced grade 1-2 TRAEs. Grade ≥3 TRAEs occurred in 19 patients (17.4%). The most frequent grade 1-2 TRAEs were digestive reactions (52.3%), neutropenia (39.4%), leukopenia (24.8%), increased ALT (21.1%), increased AST (21.1%), thrombocytopenia (19.3%) and thyroid dysfunction (18.3%). Grade ≥3 TRAEs were neutropenia (11.0%), leukopenia (6.4%), anemia (5.5%), thrombocytopenia (0.9%), and immune-related pneumonia (1.8%). All patients recovered after treatment without severe sequelae. The incidences of TRAEs are shown in **Table 4**.

**Table 4:** Treatment-related adverse events in the study cohort.

Events, N (%)	Any (N =109)	Grade 1-2	Grade ≥3
Any TRAEs	85 (78.0)	66 (60.6)	19 (17.4)
Digestive reaction	57 (52.3)	57 (52.3)	0 (0)
Leukopenia	34 (31.2)	27 (24.8)	7 (6.4)
Neutropenia	55 (50.4)	43 (39.4)	12 (11.0)
Anaemia	17 (15.6)	11 (10.1)	6 (5.5)
Thrombocytopenia	22 (20.2)	21 (19.3)	1 (0.9)
Hypertriglyceridemia	5 (4.6)	5 (4.6)	0 (0)
Hyperpotassemia	3 (2.7)	3 (2.7)	0 (0)
Thyroid dysfunction	20 (18.3)	20 (18.3)	0 (0)
Increased LDH	2 (1.8)	2 (1.8)	0 (0)
Increased blood bilirubin	12 (11.0)	12 (11.0)	0 (0)
Increased creatinine	9 (8.3)	9 (8.3)	0 (0)
Increased ALT	23 (21.1)	23 (21.1)	0 (0)
Increased AST	23 (21.1)	23 (21.1)	0 (0)
Increased CK-MB	13 (11.9)	13 (11.9)	0 (0)
Hyperglycemia	1 (0.9)	1 (0.9)	0 (0)
Immune-related pneumonia	2 (1.8)	0 (0)	2 (1.8)

TRAEs, treatment-related adverse events;LDH, Lactate Dehydrogenase; ALT, Alanine Aminotransferase; AST, Aspartate Aminotransferase; CK-MB, Creatine Kinase-MB Isoenzyme.

## Discussion

To our knowledge, this study is the first to evaluate the efficacy and feasibility of adjuvant immunotherapy after NAIC plus radical surgical resection in patients with resectable LA-ESCC. The present study revealed that the DFS and OS at 12 months were 91.0% and 99.1%, while the DFS and OS at 24 months were 81.7% and 96.8%. Subgroup analysis further demonstrated that patients with BMI ≥20 kg/m^2^, pCR, ypT0-1 stage, ypN0 stage, acquired T and TNM downstaging exhibited better short-term DFS. The majority of patients experienced TRAEs, among which the most common were digestive reactions and myelosuppression. Grade ≥3 adverse events were observed in 8 patients. These results suggest that NAIC combined with adjuvant immunotherapy is both efficacy and safe for patients with LA-ESCC.

NCRT is the standard treatment for resectable LA-ESCC, but recurrence and metastasis are still common (15, 16). Moreover, NCRT is limited in clinical practice because of its increased surgical difficulty, perioperative complications, mortality and relatively poor compliance (17, 18). Therefore, it is necessary to explore new therapeutic strategies to improve efficacy and safety for LA-ESCC. Currently, the NAIC treatment regimen has been shown to exhibit certain advantages in several clinical trials (19–23). Several prospective clinical studies reported the pCR rates ranging from 25% to 50% in patients with LA-ESCC who received NAIC (19–21). Some studies also demonstrated that NAIC had a better short-term prognosis than NCRT, with 12-month DFS rate of 86.9% to 93.4%, 12-month OS rate of 85.5% to 96%, 24-month DFS rate of 77.6% to 80.7%, and 24-month OS rates of 68.2% to 86.8% (23–28). These results suggest that NAIC is effective in resectable LA-ESCC, but it is not clear whether patients can further benefit from adjuvant treatment regimens after NAIC. Based on the purpose, we analyzed the efficacy and feasibility of adjuvant immunotherapy after NAIC in patients with LA-ESCC. First, in our study, we found that patients had a pCR rate of 20.5%. However the pCR rates reported in the ESCORT-NEO and REVO trials were approximately 35-50% (13, 14). The reason for obtaining this pCR rate may be that only the pCR status of patients who received adjuvant therapy was analyzed, and for patients without adjuvant therapy the pCR rate is unknown. We further investigated the efficacy of adjuvant immunotherapy in LA-ESCC patients. The median DFS and OS were 18.5 months and 24.0 months, respectively. In past studies, the 12 months DFS and OS rates were 68.2% and 89.4%, respectively, and the 24 months DFS and OS rates were 55.1% and 78.6%, respectively. In our study, the DFS and OS at 12 months were 91.0% and 99.1%. And the DFS and OS at 24 months were 81.7% and 96.8%. Meanwhile, the surgical R0 resection rate of patients who underwent NAIC was 100%, which was higher than that of NCT or NCRT patients, with R0 rate ranging from 81.7% to 86.6% (29, 30).

We further performed subgroup analyses of DFS and OS. Previous studies have shown that patients with a high BMI have a better prognosis in LA-ESCC (31). We found that superior 24-month DFS was associated with BMI ≥20 kg/m^2^ (87.1% vs 62.0%, P=0.034), indicating that the nutritional status of patients is closely linked with the prognosis of cancer patients. Additionally, research in the past reported that patients with postoperative pCR achieved better survival benefit than those with non-pCR (16, 23, 32). Similarly, we demonstrated that patients who achieved pCR and received adjuvant immunotherapy acquired better 12-month (100% vs 88.6%, P<0.001) and 24-month (100% vs 77.3%, P<0.001) DFS than those with non-pCR. Furthermore, Superior 12- and 24-month DFS rates were observed in patients with ypT0-1 versus ypT2-4 (12-month: 98.1% vs 84.6%, P=0.008, 24-month: 95.4% vs 70.7%, P<0.001), and ypN0 versus ypN+ (12-month: 96.9% vs 83.1%, P=0.019, 24-month: 88.9% vs 72.2%, P=0.042). These findings suggested that patients receiving adjuvant immunotherapy at an earlier stage may achieve short-term survival benefits. However, further comparisons with patients not receiving adjuvant therapy are needed to assess long-term prognosis. Moreover, better 12- and 24-months DFS rate were showed in patients who obtained T (12-month: 96.2% vs 78.3%, P=0.018, 24-month: 92.8% vs 56.0%, P<0.001) and TNM (12-month: 96.5% vs 84.8%, P=0.033, 24-month: 90.5% vs 72.5%, P=0.021) downstaging. This implies that the effectiveness of NAIC, and patients who received NAIC may further benefit from adjuvant immunotherapy.

The association between PD-L1 expression levels and the prognosis of patients with adjuvant immunotherapy was evaluated in the present study. PD-L1 is expressed by tumor cells to help them evade the host immune response and is considered a poor prognostic biomarker for patient survival (33). The expression of PD-L1 protein in the TME is regarded as a predictive biomarker of anti-PD-1 therapy efficacy in cancer treatment (11). Previous studies of ICIs with first-line chemotherapy in patients with advanced esophageal cancer and gastroesophageal junction cancer have demonstrated that survival benefits were enhanced in patients of high PD-L1 expression level according to CPS (34, 35). In our study, patients with CPS ≥10 had a better 12- and 24-month DFS rates (100% vs 83.3%). However, as many patients in our study did not undergo PD-L1 testing, no statistically significant difference could be observed between the two groups. Therefore, future studies with larger sample sizes are needed to further validate the association between PD-L1 expression and the efficacy of neoadjuvant and adjuvant immunotherapy in LA-ESCC.

Safety remains an issue that requires close attention in the long-term treatment of cancer. Several clinical trials have shown no significant difference in TRAEs between immunotherapy and immunotherapy combined with chemotherapy in LA-ESCC (36), which indicates an acceptable safety profile for adjuvant immunotherapy plus chemotherapy. During the application of immunotherapy and chemotherapy, the most common serious TRAEs included myelosuppression (37–39). Similar results were obtained in our study, which found that 85 patients had TRAEs. The most common TRAEs are digestive tract reactions, myelosuppression, hepatic function impairment and thrombocytopenia. Among these, grade ≥3 TRAEs primarily consisted of hematologic toxicities, particularly myelosuppression. Similar to the results of previous studies, it can all be proved that the addition of immunotherapy does not significantly increase adverse reactions. Overall, the combination of NAIC and adjuvant immunotherapy was deemed safe.

This study confirmed the efficacy and safety of NAIC combined with adjuvant immunotherapy in patients with LA-ESCC, but it still has several critical limitations. First, because this was a retrospective study, no control group was included, and direct comparisons with other standard treatment regimens could not be made. It is therefore difficult to determine whether the observed survival benefits and safety are superior to existing treatment strategies. Future studies should incorporate randomized controlled trials (RCTS) to validate the efficacy and safety of this regimen. Second, the sample size of this study was relatively small, and larger, multicenter prospective studies are needed to provide more reliable treatment outcomes to evaluate and validate. Third, the study population exhibited heterogeneity in treatment regimens because they received different immunotherapeutic agents or combination regimens. This variability may have influenced efficacy. Future studies should explore the impact of specific treatment regimens in a more standardized manner to determine optimal treatment. Finally, the median follow-up period was 24 months, which is relatively short for assessing long-term survival and delayed adverse events. A longer follow-up period is critical for assessing the durability of treatment effects and identifying any delayed toxicities associated with the regimen. At the same time, this resulted in a limited number of DFS and OS outcome events, which made multivariate Cox regression analyses unreliable, so we performed only univariate analyses. Future studies should explore different perioperative therapies to determine the most effective regimen for improving survival outcomes in patients with LA-ESCC. Despite these limitations, our findings provide preliminary evidence to support NAIC combined with adjuvant immunotherapy as a promising treatment strategy for LA-ESCC. Further large-scale prospective clinical trials are needed to confirm its long-term benefits and to identify the optimal patient population for this approach.

## Conclusions

Adjuvant immunotherapy following NAIC is a relatively new treatment regimen for resectable LA-ESCC, which improves the survival rate in patients with operable LA-ESCC without significantly increasing adverse reaction incidence. For patients with LA-ESCC who undergo NAIC followed by surgical resection and adjuvant immunotherapy, having a high BMI, achieving pCR, ypT0-1 and ypN0, and achieving tumor downstaging may be key factors influencing prognosis.

## Data Availability

The raw data supporting the conclusions of this article will be made available by the authors, without undue reservation.

## Supplementary material

Supplementary Figure 1 Kaplan-Meier survival analysis of OS between BMI ≥20 kg/m^2^ and BMI <20 kg/m^2^ **(A)**, between pCR and non-pCR **(B)**, between ypT0-1 and ypT2-4 **(C)**, between ypN0 and ypN+ **(D)**, between T downstaging and without T downstaging **(E)**, and between TNM downstaging and without TNM downstaging **(F)**. DFS, disease-free survival; OS, overall survival; BMI, body mass index; pCR, pathological complete response.

Supplementary Figure 2 Kaplan-Meier survival analysis of DFS **(A)** and OS **(B)** between clinical N0 and N+, DFS **(C)** and OS **(D)** between clinical stage II and stage III-IV, and DFS **(E)** and OS **(F)** between N downstaging and without N downstaging. DFS, disease-free survival; OS, overall survival.

Supplementary Figure 3 Kaplan-Meier survival analysis of DFS **(A)** and OS **(B)** between >2 cycle and ≤2 cycle of NACI, and DFS **(C)** and OS **(D)** between >2 cycle and ≤2 cycle of adjuvant immunotherapy. DFS, disease-free survival; OS, overall survival; NAIC, neoadjuvant immunotherapy with chemotherapy.

Supplementary Figure 4 Kaplan-Meier survival analysis of DFS **(A)** and OS **(B)** between interval time >4 weeks and ≤4 weeks of NAIC to surgery, and DFS **(C)** and OS **(D)** between interval >4 weeks and interval ≤4 weeks of surgery to adjuvant immunotherapy. DFS, disease-free survival; OS, overall survival; NAIC, neoadjuvant immunotherapy with chemotherapy.

Supplementary Figure 5 Kaplan-Meier survival analysis of DFS **(A)** and OS **(B)** between combined with chemotherapy and without chemotherapy in adjuvant therapy, DFS **(C)** and OS **(D)** between combined with radiotherapy and without radiotherapy in adjuvant therapy. DFS, disease-free survival; OS overall survival.
