# Supplementary figures and images for: The efficacy and safety of adjuvant immunotherapy after neoadjuvant immunotherapy combined with chemotherapy in locally advanced resectable esophageal squamous cell carcinoma: a real−world study
Source: Front Immunol. 2025 May 21;16:1555756. doi: 10.3389/fimmu.2025.1555756 (PMC12133854; doi:10.3389/fimmu.2025.1555756)

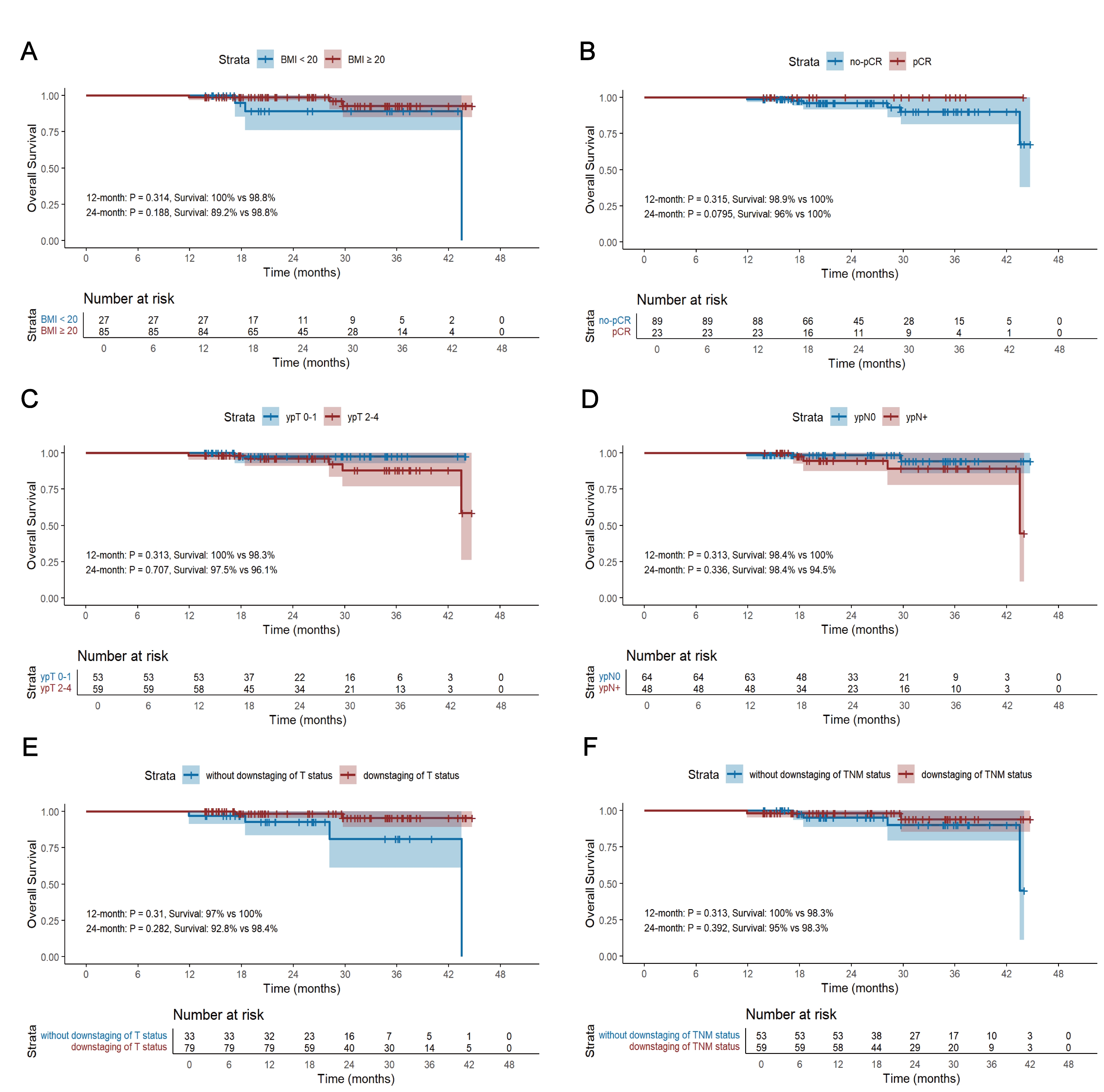

Supplement: Supplementary Figure 1 — Kaplan-Meier survival analysis of OS between BMI ≥20 kg/m2 and BMI <20 kg/m2 (A), between pCR and non-pCR (B), between ypT0-1 and ypT2-4 (C), between ypN0 and ypN+ (D), between T downstaging and without T downstaging (E), and between TNM downstaging and without TNM downstaging (F). DFS, disease-free survival; OS, overall survival; BMI, body mass index; pCR, pathological complete response. [file Image1.jpeg]

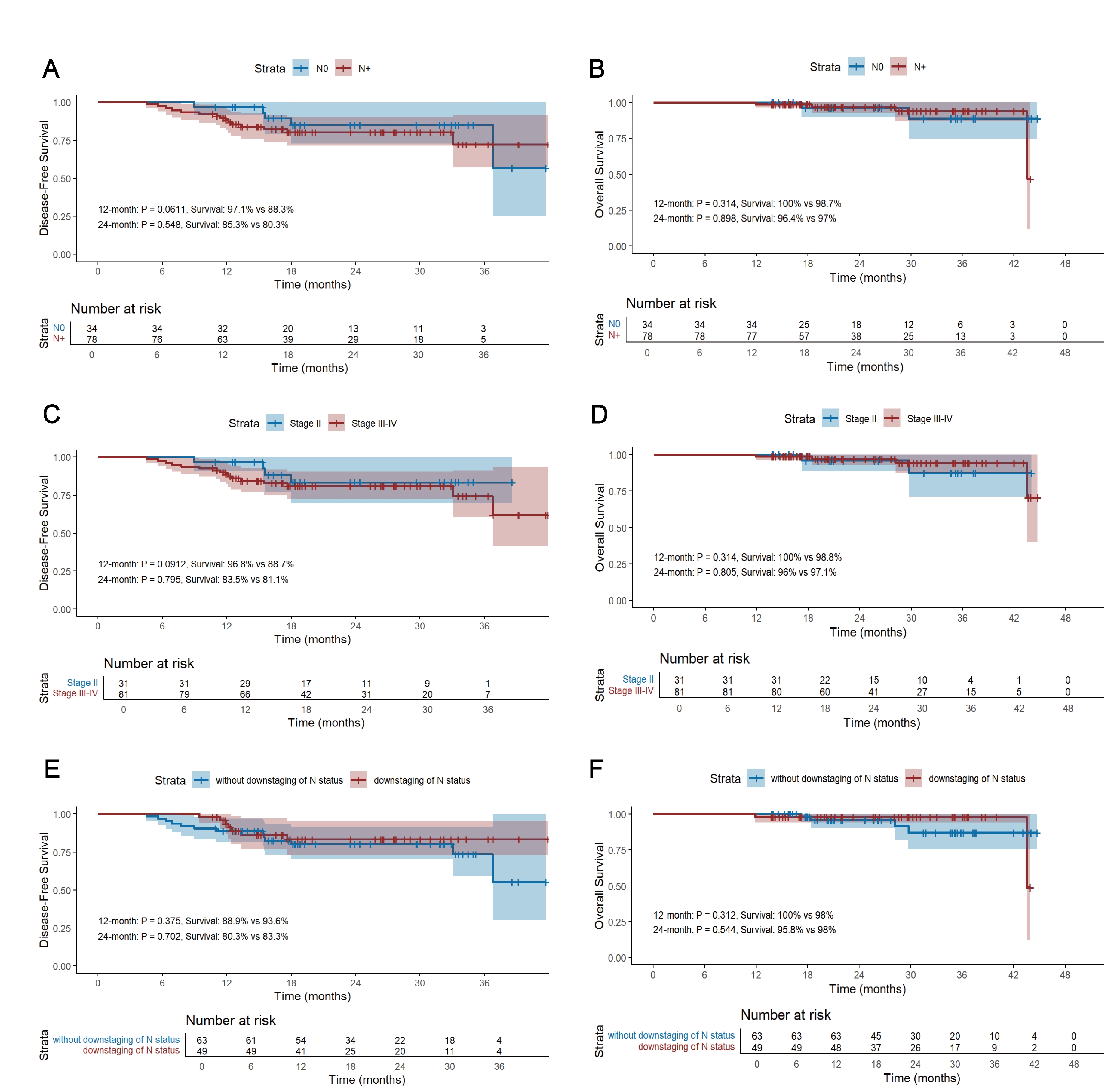

Supplement: Supplementary Figure 2 — Kaplan-Meier survival analysis of DFS (A) and OS (B) between clinical N0 and N+, DFS (C) and OS (D) between clinical stage II and stage III-IV, and DFS (E) and OS (F) between N downstaging and without N downstaging. DFS, disease-free survival; OS, overall survival. [file Image2.jpeg]

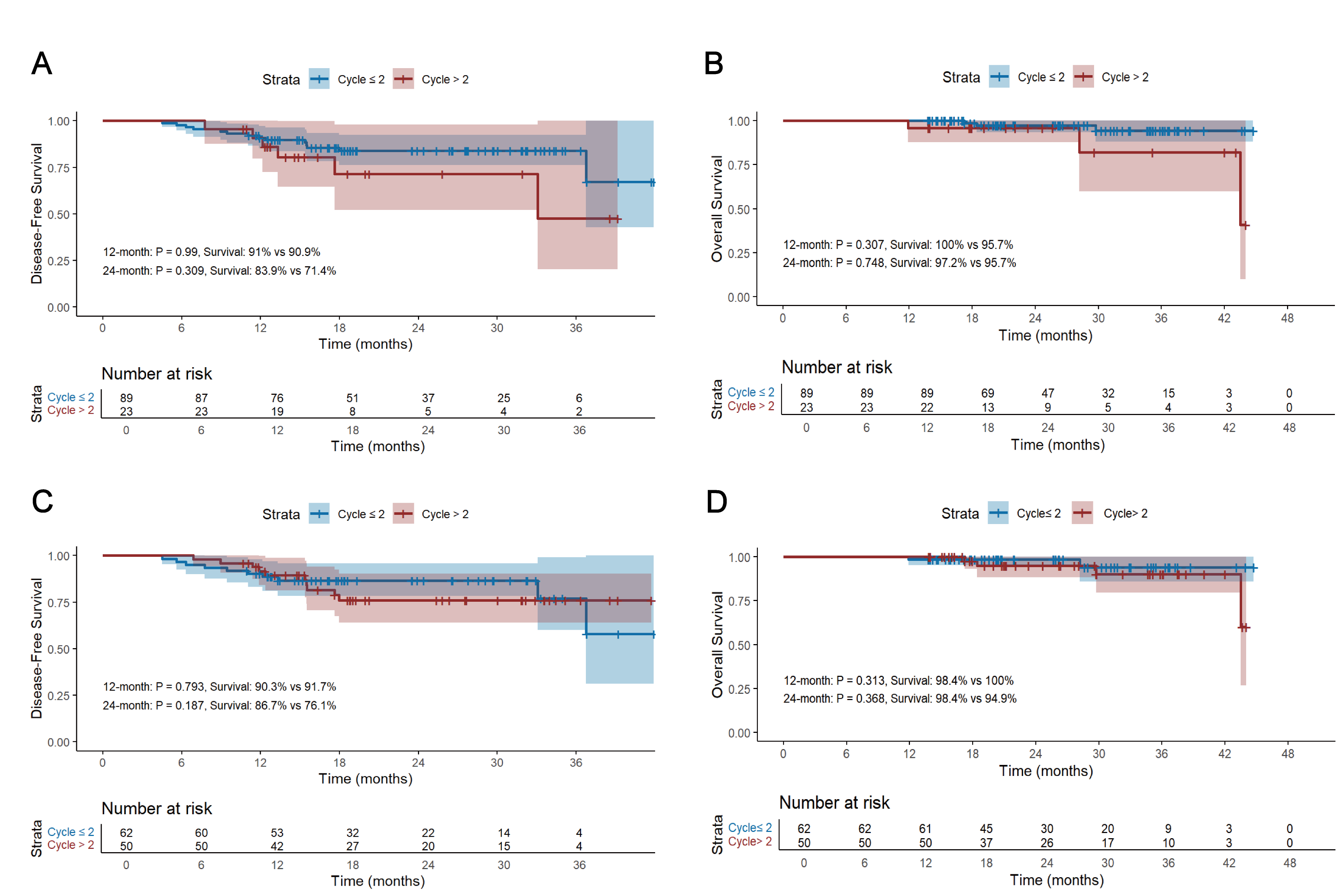

Supplement: Supplementary Figure 3 — Kaplan-Meier survival analysis of DFS (A) and OS (B) between >2 cycle and ≤2 cycle of NACI, and DFS (C) and OS (D) between >2 cycle and ≤2 cycle of adjuvant immunotherapy. DFS, disease-free survival; OS, overall survival; NAIC, neoadjuvant immunotherapy with chemotherapy. [file Image3.tif]

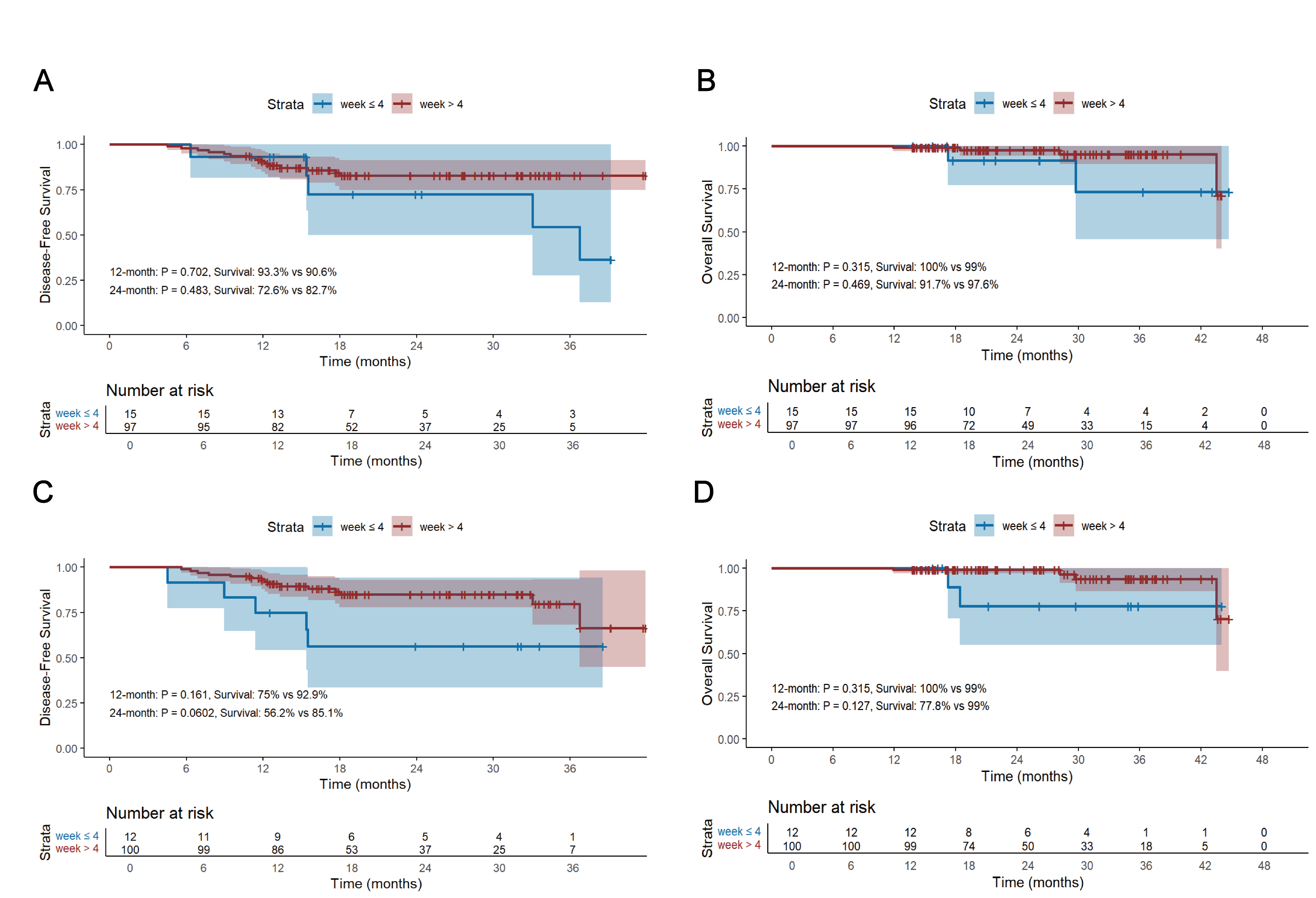

Supplement: Supplementary Figure 4 — Kaplan-Meier survival analysis of DFS (A) and OS (B) between interval time >4 weeks and ≤4 weeks of NAIC to surgery, and DFS (C) and OS (D) between interval >4 weeks and interval ≤4 weeks of surgery to adjuvant immunotherapy. DFS, disease-free survival; OS, overall survival; NAIC, neoadjuvant immunotherapy with chemotherapy. [file Image4.tif]

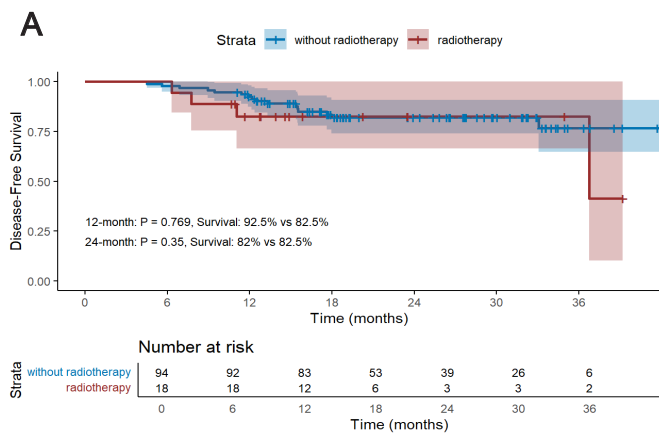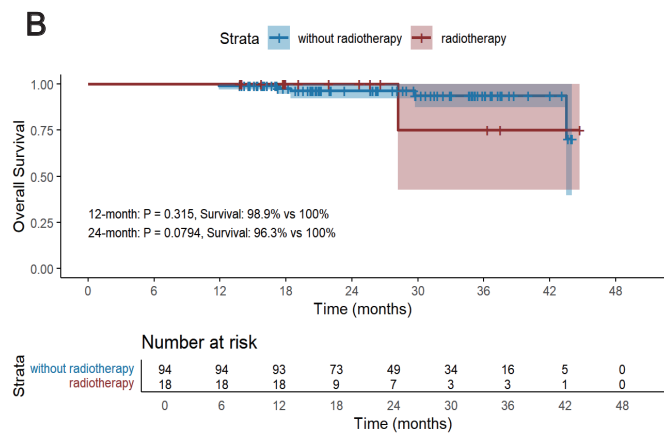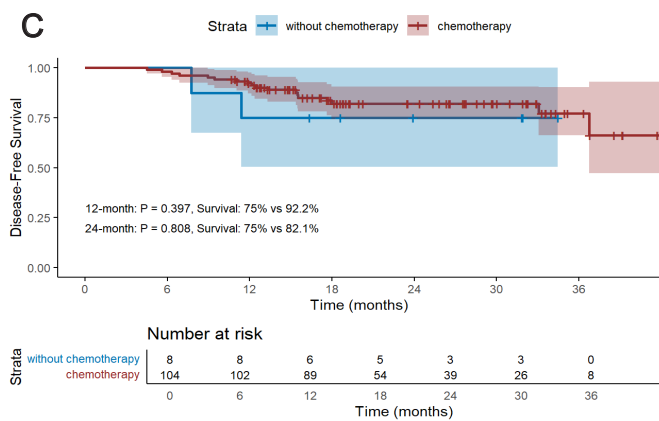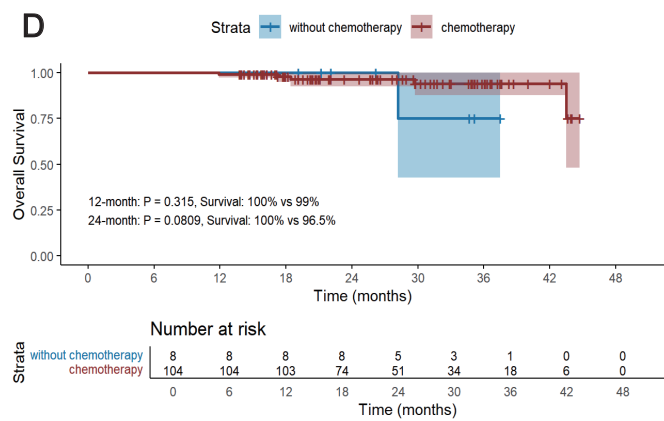

Supplement: Supplementary Figure 5 — Kaplan-Meier survival analysis of DFS (A) and OS (B) between combined with chemotherapy and without chemotherapy in adjuvant therapy, DFS (C) and OS (D) between combined with radiotherapy and without radiotherapy in adjuvant therapy. DFS, disease-free survival; OS overall survival. [file DataSheet1.pdf]
